# A psychometric study of the Family Resilience Assessment Scale among families of children with autism spectrum disorder

**DOI:** 10.1186/s12955-019-1117-x

**Published:** 2019-03-12

**Authors:** Emily Gardiner, Louise C. Mâsse, Grace Iarocci

**Affiliations:** 1 BC Children’s Hospital Research Institute, 950 West 28th Avenue, Vancouver, BC V5Z 4H4 Canada; 2 Department of Pediatrics, University of British Columbia, 4480 Oak Street, Vancouver, BC V6H 3V4 Canada; 3 School of Population and Public Health, University of British Columbia, 2206 East Mall, Vancouver, BC V6T 1Z3 Canada; 4 Department of Psychology, Simon Fraser University, 8888 University Drive, Burnaby, BC V5A 1S6 Canada

**Keywords:** Autism spectrum disorder, Family resilience, Assessment, Factor analysis, Family quality of life

## Abstract

**Background:**

The family system represents a critical context within which children develop. Although raising a child with a disability may represent a challenge to this dynamic system, research demonstrates that families have the capacity to demonstrate both maladaptation and resilience in the face of related stressors. In the current study, we examined the psychometric properties of the Family Resilience Assessment Scale (FRAS) among families of children with autism spectrum disorder (ASD). This tool is the only measure of family resilience that seeks to identify within-family protective factors, including the extent to which they rely on adaptive belief systems, organizational patterns, and communication processes. Identifying protective processes utilized by those who show resilience is critical within both clinical practice and research, as it aligns with a strength-based perspective that builds on what families are doing well.

**Methods:**

Participants included 174 caregivers of individuals with ASD (84% mothers). Caregivers completed the FRAS, as well as the Beach Center Family Quality of Life Scale. The 54-item FRAS was submitted to an exploratory factor analysis, using the iterated principal factor method with a promax rotation.

**Results:**

Fifty-one items across 3 factors (Family Communication and Problem Solving, Utilizing Social and Economic Resources, Family Spirituality) were retained, explaining 52% of the total variance. The final scale demonstrated convergent validity with the Family Quality of Life assessment tool.

**Conclusions:**

It is our hope that identifying the optimal scale structure will encourage other researchers to utilize this measure with families of children with ASD, thus continuing to advance the study of family resilience within this unique context.

## Background

The study of resilience emerged from early work that identified groups of children who demonstrated a remarkable ability to not only recover, but flourish despite early conditions characterized by extreme adversity (e.g., [1–3]). In such work, the family was often viewed as a primary mechanism of pathology, and something that resilient children were able to successfully overcome. Work to expand the construct of resilience has increasingly acknowledged that although the family environment may impose constraints that place a child’s optimal development at risk, it can also serve an important protective function [4]. In particular, strong inter-family relationships have been shown to play important buffering roles in the face of different circumstances of adversity, such as divorce [5], chronic illness [6], and disability [7]. This has contributed to the proliferation of a strength-based perspective in research and practice, in which the family is not viewed as disordered, but as capable [8]. The family is considered to be a dynamic system that holds the capacity to demonstrate both maladaptation and resilience in the face of encountered stressors [9]. By identifying critical protective processes utilized by those who show resilience, professionals working with at-risk families can encourage positive appraisal and coping patterns that promote continued adaptation [10, 11].

Raising a child with autism spectrum disorder (ASD) represents one circumstance that may place the family at risk. Upon receiving a diagnosis, families must re-negotiate established roles and adjust their expectations for child rearing within this new and uncharted context [12]. Although this situation may present considerable challenge, with research indicating that many families experience heightened stress [13] and poor quality of life [14], qualitative research highlights families’ tremendous capacity for resilience [15–18].

Various models of family resilience have been put forth, primarily by McCubbin and colleagues (e.g., Double ABCX, Family Adjustment and Adaptation Response, T-Double ABCX, Resiliency Model of Family Adjustment and Adaptation; see Nichols [4] for a review), that theorize about the interactional nature of stressors, family perceptions, and protective factors in the facilitation of resilience. These theories, however, do not suggest what those protective factors might be. Walsh has written extensively about critical family processes, as outlined within her framework that conceptualizes resilience as more than surviving, but as emerging strengthened and more resourceful [19–22]. Walsh’s perspective aligns with findings from qualitative research with families of children with ASD, in which participants’ descriptions suggest that their experiences are more than a ‘bouncing back’ from adversity, but instead reflect a process of growth. For example, in Bayat’s [15] study, one parent described a strengthened marriage, with another stating: “Autism has made us stronger and more cohesive” (p. 709).

Walsh [20, 21] suggests that family resilience involves three broad processes, each containing three sub-processes: Family Belief Systems that allow individuals to find meaning within adversity, maintain an optimistic outlook, and to have strong spiritual beliefs; Organizational Patterns that are flexible, connected, and include access to necessary social and economic resources; and Communication Processes characterized by clarity, open emotional expression, and collaborative problem solving. The Family Resilience Assessment Scale (FRAS) [23], which is based on Walsh’s model, includes 6 subscales, which are meant to reflect Walsh’s 9 processes. Table 1 presents the conceptual alignment between the 6 FRAS subscales and Walsh’s 9 processes.

**Table 1:** Walsh’s Family Resilience Processes and Family Resilience Assessment Scale (FRAS) Subscales

Walsh’s Model	FRAS Subscales
Belief Systems	
Making meaning of adversity	Ability to Make Meaning from Adversity *(3 items)*
Positive outlook	Maintaining a Positive Outlook *(6 items)*
Transcendence and spirituality	Family Spirituality *(4 items)*
Organizational Patterns	
Flexibility	Family Connectedness *(6 items)*
Connectedness	Utilizing Social and Economic Resources *(8 items)*
Social and economic resources	
Communication Processes	
Clear, consistent messages	Family Communication and Problem Solving *(27 items)*
Open emotional expression	
Collaborative problem solving	

*Note. FRAS* Family Resilience Assessment Scale

The FRAS has received considerable attention, and has been used to examine family resilience in various contexts, including among international adoptees [24], vocational rehabilitation clients [25], as well as among families of children with developmental disabilities [26], including epilepsy [27], attention deficit hyperactivity disorder [28] and ASD [29–31]. Moreover, validation studies have been conducted with participants from Singapore [27], Turkey [32], and China [33], though none have included caregivers of children with disabilities.

Conceptually, the FRAS is particularly appropriate to study family life when a child is diagnosed with ASD, as the subscales reflect processes that have been identified within qualitative research as important for family adaptation. For example, research with families of children with ASD has shown that positive appraisal and humour are important protective factors [16], and highlights the importance of family environments characterized by mutually supportive relationships [15, 17]. Others report on the centrality of family spirituality [15], as well as families’ reliance on social support networks [16, 17, 34]. As the English version of the scale has not been validated with caregivers beyond the original scale development, we do not know whether the FRAS’ proposed 6-factor model is appropriate for families of children with ASD.

In the present study, we examined the psychometric properties of the 6 scales included within the FRAS among families who care for a child with ASD to determine whether the structural properties of the scales hold. In addition, this study examined whether the FRAS was associated with the Beach Center Family Quality of Life Scale [35]. It is our hope that identifying the optimal scale structure will encourage other researchers in the field to utilize this measure with families of children with ASD, thus continuing to advance the study of family resilience within this unique context.

## Methods

### Participants

Data were examined from 174 caregivers of individuals with ASD who had participated in a larger study exploring family quality of life and resilience. Caregivers were mostly mothers (83.9%) and represented a range of family ethnicities. Most were married, had received some kind of formal education post high school, and had high family incomes, with the median reported income range being $80,000 - $109,999. Almost half the sample (42.5%) indicated their family had experienced a significant life event (e.g., divorce, death, move, job loss) in the previous 6 months, most frequently endorsing death of a family member or close friend (24.3%), move (23.0%), family breakup (21.6%) (i.e., divorce or separation), or family illness or injury (17.6%). Informed consent was obtained by all individual participants included in the study, and the study received approval from the University Research Ethics Board. See Table 2 for demographic characteristics of the sample.

**Table 2:** Sample Demographic Characteristics

	*n* (%)	*M* (*SD*)
Respondent		
Mother	146 (83.9)	
Father	26 (14.9)	
Other	2 (1.1)	
Respondent Age (years)		45.32 (7.64)
20–29 years	1 (.6)	
30–39 years	45 (25.9)	
40–49 years	83 (47.7)	
50–59 years	38 (21.8)	
60–69 years	7 (4.0)	
Family Ethnicity		
Aboriginal	2 (1.1)	
African	2 (1.1)	
Asian	73 (42.0)	
Canadian	58 (33.3)	
European	26 (14.9)	
Latin American	1 (0.6)	
Multiple Identified	57 (32.8)	
Marital Status		
Married or Common Law	137 (78.7)	
Divorced or Separated	25 (14.4)	
Widowed	2 (1.1)	
Never Married	10 (5.7)	
Respondent Education		
High School	19 (10.3)	
Professional Diploma	39 (22.4)	
Undergraduate Degree	60 (34.5)	
Graduate Degree	42 (24.1)	
Other	13 (7.5)	
Family Income		
< $20,000	8 (4.6)	
$21,000 - $49,999	32 (18.4)	
$50,000 - $79,999	38 (21.8)	
$80,000 - $109,999	42 (24.1)	
> $110,000	54 (31.0)	
Child Age		11.93 (5.84)
2–5 years	26 (14.9)	
6–18 years	127 (73.0)	
19–35 years	21 (12.1)	
Child Gender		
Male	148 (85.1)	
Female	20 (11.5)	

#### Diagnostic confirmation

All individuals with ASD had received a standardized clinical diagnosis of ASD from a qualified paediatrician, registered doctoral-level psychologist, or psychiatrist associated with the provincial government-funded autism assessment network, or through a qualified private clinician. All diagnoses were based on the *Diagnostic and Statistical Manual of Mental Disorders* (*DSM*) [36, 37] and confirmed using the Autism Diagnostic Interview–Revised (ADI-R) [38] and Autism Diagnostic Observation Schedule (ADOS) [39], both of which are gold standard tools of ASD diagnostic assessment. As the ASD diagnosis is tied directly to substantial provincial funding programs, British Columbia has instituted standardized diagnostic practices. All individuals are required to be diagnosed by ADOS- and ADI-R-trained clinicians who use these tools and clinical judgment to make the diagnosis. This also pertains to individuals who have been diagnosed in a different province or country, as they are required to be re-diagnosed upon their arrival to British Columbia using these assessment standards.

### Measures

#### Family resilience

The FRAS [23] includes 54 items and 6 scales (see Table 1), to which respondents are asked to rate the extent that each item describes their family based on a 4-point Likert-type scale ranging from ‘Strongly Disagree’ (1) to ‘Strongly Agree’ (4). Responses are summed, with higher scores indicating greater resilience. The instrument has been shown to demonstrate good internal consistency across the total and subscale scores (alpha = .70–.96) [23]. Subscales on the FRAS also demonstrate strong convergent validity with subscales of the Family Assessment Device (FAD) [40], a well-established tool assessing structural, organizational, and transactional aspects of family life [41].

#### Family quality of life

The Beach Center Family Quality of Life (FQOL) Scale [35] assesses FQOL across five domains: Family Interaction, Parenting, Emotional Well-Being, Physical/Material Well-Being, and Disability-Related Support. This measure includes 25 questions with responses based on a 5-point rating scale ranging from ‘Very Dissatisfied’ (1) to ‘Very Satisfied’ (5). Domain scores are determined by calculating the mean rating of domain-relevant items (ranging from 1 to 5). An overall score can also be calculated by averaging all item ratings, with higher scores signifying greater quality of life satisfaction. The scale is internally consistent (alpha values ranged from 0.72 to 0.86, and was 0.92 for the overall score in this study), and has demonstrated concurrent validity with other family scales (specifically the Family APGAR [42] and Family Resources Scale [43]; see also [44]), and test-retest reliability for each subscale, as assessed 3 months apart, ranged from .60–.77 [35]. As the FQOL Scale is a well-established tool that has been used extensively among families of children with ASD [13, 34, 45–49], we expected that particular FQOL Scale subscales (e.g., Family Interaction) would be positively associated with FRAS subscales (e.g., Family Communication and Problem Solving). Thus, the FQOL Scale was chosen to establish convergent validity.

### Statistical analyses

All data analyses were conducted using Stata, Version 14.2. To assess the structural properties of the FRAS among our sample of families of individuals with ASD, the 54-item measure was submitted to a Confirmatory Factor Analysis (CFA). A CFA was conducted on the proposed 6-factor model, and model fit was assessed with various indices, including Root Mean Squared Error of Approximation (RMSEA), Comparative Fit Index (CFI), Tucker-Lewis Index (TLI), and Standardized Root Square Mean Residual (SRMR). Cut-off criteria as recommended by Hu and Bentler [50] were utilized, such that for the RMSEA, a value up to .06 with a 90% confidence interval (CI) less than .08 was considered acceptable [51]. Cut-off values of .95 were utilized for the CFI and TLI, and values up to .08 were considered acceptable for the SRMR. The maximum likelihood estimation procedure was used as the data did not severely violate the normality assumption. As the 6-factor structure did not hold as hypothesized and evaluation of the modification indices did not result in modification that significantly improved the fit of the solution, the 54 FRAS items were submitted to an exploratory factor analysis (EFA). The maximum likelihood method of extraction was first utilized; however, the solution did not converge due to the presence of a Heywood case. As such, the iterated principal factor method with a promax rotation was utilized to identify the number of latent variables represented within the FRAS. Both the eigenvalues rule of 1 and percentage of variance explained by each factor (explaining at least 5 to 10% of the total variance) were evaluated to determine the number of factors retained with the EFA procedure. In addition, the solution was conceptually evaluated.

## Results

### Structural properties of the FRAS

The proposed 6-factor model, which includes 54 measured variables, was submitted to a CFA. The overall fit of the model was not optimal [χ^2^ (df = 1362) = 2401.53; RMSEA = .07; 90% CI = .06–.07; CFI = .81; TLI = .80; SRMR = .08], as the CFI and TLI were both well below recommended levels. Moreover, examination of the modification indices highlighted significant issues with the existing factor structure, and significant cross-loading among many variables onto other factors, leading us to re-evaluate the fit of the overall scale. As any attempts to include some of these cross-loadings did not result in improved model fit, an EFA using the iterated principal factor method of extraction was run on the data. Evaluation of the eigenvalues and percentage of variance explained by each factor suggested that a 3-factor solution be retained. The initial solution identified one item that did not load on any factors (factor loadings less than .30 in absolute value), and this item was therefore dropped (“We feel taken for granted by family members”). When the EFA was rerun, two additional items did not load on any of the three factors and were also removed (“We keep our feelings to ourselves”; “We seldom listen to family members’ concerns or problems”). See Tables 3 and 4 for item and subscale descriptives. Item skewness ranged from −.76–.97. Item kurtosis ranged from − 1.00-3.15, and 78% of the items had kurtosis values less than |1.0|. The EFA on the remaining 51-items (FRAS-ASD) suggested that a 3-factor solution be retained and the total solution explained 52.2% of the total variance. For this solution, there were no cross-loadings. The three factors conceptually measured the following concepts: ‘Family Communication and Problem Solving’ (Factor 1), ‘Utilizing Social and Economic Resources’ (Factor 2), and ‘Family Spirituality’ (Factor 3). Factors 1 to 3 explained 31, 14.7, and 6.5% of the total variance, respectively. The EFA solution is shown in Table 4. Although Sixbey [23] indicates that subscale scores are comprised of the total of relevant item responses (i.e., additive), we report average scores, as this is more amenable for comparing family’s perceived resilience across subscales. The correlations among the 3 factors are presented in Table 5.

**Table 3:** Subscale Descriptive Statistics and Internal Reliabilities for 51-item Family Resilience Assessment Scale (FRAS-ASD)

FRAS-ASD Factor Scale	Cronbach’s α	*M* (*SD*)	Skew	Kurtosis
Family Communication and Problem Solving	.96	3.12 (.40)	−.01	.46
Utilizing Social and Economic Resources	.88	2.78 (.51)	−.18	.08
Family Spirituality	.92	2.11 (.90)	.57	−.63

**Table 4:** Descriptive Statistics and Standardized Factor Loadings for 51-item Family Resilience Assessment Scale (FRAS-ASD)

Factors and Items	Standardized Factor Loadings					
	Factor 1	Factor 2	Factor 3	*M* (*SD*)	Range	ITC
Factor 1: Family Communication and Problem Solving						
1. Our family structure is flexible to deal with the unexpected.	.49	.03	−.02	3.12 (.65)	1–4	.49
3. The things we do for each other make us feel a part of the family.	.44	.12	.04	3.32 (.56)	1–4	.48
4. We accept stressful events as part of life.	.57	−.02	−.00	3.20 (.65)	1–4	.55
5. We accept that problems occur unexpectedly.	.59	−.06	.02	3.28 (.52)	2–4	.56
6. We all have input into major family decisions.	.65	−.13	.00	3.09 (.71)	1–4	.59
7. We are able to work through pain and come to an understanding.	.77	−.05	.03	3.11 (.63)	1–4	.74
8. We are adaptable to demands placed on us as a family.	.74	.02	−.03	3.10 (.57)	1–4	.73
9. We are open to new ways of doing things in our family.	.59	−.13	.12	3.10 (.63)	2–4	.52
13. We believe we can handle our problems.	.63	.16	−.08	2.96 (.62)	1–4	.68
14. We can ask for clarification if we do not understand each other.	.72	−.07	−.06	3.11 (.61)	1–4	.67
15. We can be honest and direct with each other in our family.	.74	−.16	.04	3.17 (.64)	1–4	.66
16. We can blow off steam at home without upsetting someone.	.53	.11	−.03	2.55 (.72)	1–4	.56
17. We can compromise when problems come up.	.83	−.11	.06	3.04 (.58)	1–4	.77
18. We can deal with family differences in accepting a loss.	.65	.08	−.02	3.07 (.54)	1–4	.67
20. We can question the meaning behind messages in our family.	.50	.11	−.01	2.97 (.62)	1–4	.53
21. We can solve major problems.	.74	.06	.02	3.10 (.65)	1–4	.75
22. We can survive if another problem comes up.	.63	.21	−.07	3.22 (.64)	1–4	.70
23. We can talk about the way we communicate in our family.	.76	−.06	.00	3.03 (.69)	1–4	.72
24. We can work through difficulties as a family.	.82	−.08	.08	3.18 (.58)	1–4	.77
25. We consult with each other about decisions.	.78	−.04	−.02	3.12 (.72)	1–4	.75
26. We define problems positively to solve them.	.71	−.06	.12	2.90 (.65)	1–4	.67
27. We discuss problems and feel good about the solutions.	.75	−.05	.14	2.93 (.62)	1–4	.71
28. We discuss things until we reach a resolution.	.80	−.06	.09	2.86 (.72)	1–4	.76
29. We feel free to express our opinions.	.75	−.08	−.10	3.14 (.66)	1–4	.70
30. We feel good giving time and energy to our family.	.57	.13	.01	3.32 (.63)	1–4	.61
33. We feel we are strong in facing big problems.	.66	.02	−.06	3.10 (.62)	1–4	.66
35. We have the strength to solve our problems.	.66	.02	−.13	3.16 (.53)	2–4	.66
38. We learn from each other’s mistakes.	.56	.04	−.04	3.15 (.52)	2–4	.57
39. We mean what we say to each other in our family.	.49	.13	−.02	3.17 (.50)	2–4	.54
43. We share responsibility in the family.	.56	−.04	.02	3.06 (.61)	1–4	.53
44. We show love and affection for family members.	.51	.06	−.00	3.51 (.55)	2–4	.53
45. We tell each other how much we care for one another.	.48	.17	−.06	3.40 (.65)	2–4	.54
48. We trust things will work out even in difficult times.	.45	.23	.06	3.21 (.61)	1–4	.53
49. We try new ways of working with problems.	.53	−.06	−.05	3.09 (.48)	2–4	.50
50. We understand communication from other family members.	.53	.05	−.04	3.00 (.47)	1–4	.54
51. We work to make sure family members are not emotionally or physically hurt.	.47	.13	−.14	3.36 (.57)	1–4	.51
Factor 2: Utilizing Social and Economic Resources						
2. Our friends value us and who we are.	.15	.46	.03	3.22 (.63)	1–4	.49
10. We are understood by other family members.	.25	.35	.06	2.55 (.82)	1–4	.44
11. We ask neighbors for help and assistance.	−.09	.56	.17	2.03 (.83)	1–4	.56
19. We can depend upon people in this community.	−.17	.88	.09	2.60 (.83)	1–4	.77
31. We feel people in this community are willing to help in an emergency.	.03	.81	.04	2.83 (.81)	1–4	.76
32. We feel secure living in this community.	.12	.65	−.13	3.11 (.65)	1–4	.62
36. We know there is community help if there is trouble.	−.09	.77	−.02	2.77 (.82)	1–4	.66
37. We know we are important to our friends.	.12	.57	−.02	2.99 (.64)	1–4	.56
41. We receive gifts and favors from neighbors	−.15	.65	.20	2.27 (.88)	1–4	.61
46. We think this is a good community to raise children	.06	.61	−.04	3.20 (.64)	1–4	.57
47. We think we should not get too involved with people in this community.^a^	−.15	.62	−.13	3.02 (.64)	1–4	.49
Factor 3: Family Spirituality						
12. We attend church/synagogue/mosque services.	.01	.03	.91	1.96 (1.06)	1–4	.87
34. We have faith in a supreme being.	.14	−.04	.68	2.68 (1.01)	1–4	.66
40. We participate in church activities.	.01	.05	.93	1.98 (1.03)	1–4	.89
42. We seek advice from religious advisors.	.01	.06	.89	1.82 (.94)	1–4	.84

Items included with permission of Sixbey

*Note. ITC* Item-total correlation

^a^Indicates item is reverse-scored

**Table 5:** Correlations among the 51-item Family Resilience Assessment Scale (FRAS-ASD) Factors

FRAS-ASD Factor Scale	1	2	3
1. Family Communication and Problem Solving	–		
2. Utilizing Social and Economic Resources	.40	–	
3. Family Spirituality	−.02	.12	–

### Convergent validity

The Family Communication and Problem Solving subscale showed a strong and significant association with the Beach Center FQOL Scale’s Family Interaction subscale (*r* = .70, *p* < .001), which assesses how well respondents feel their family can solve problems, talk openly and show affection, as well as handle unexpected events. The Utilizing Social and Economic Resources subscale was strongly related to the FQOL Emotional Well-Being (*r* = .63, *p* < .001) and Physical/Material Well-Being (*r* = .41, *p* < .001) subscales. The former assesses the extent to which respondents feel they can rely on family members for support, as well as access help outside the family. The Physical/Material Well-Being subscale includes items about access to transportation and medical care, but also includes an item about feeling safe in the community. As expected, the FRAS-ASD Family Spirituality subscale was not significantly correlated with any of the FQOL subscales. With the exception of Family Spirituality, associations between the FRAS-ASD and FQOL subscales not noted here were in the medium-to-large range [52] (see Table 6).

**Table 6:** Correlations Between FRAS-ASD and Beach Center FQOL Scale Subscales

FRAS-ASD Scale	Beach Center FQOL Scale					
	FI	PAR	EWB	PMWB	DRS	FQOL Ttl
FCPS	.70^*^	.52^*^	.35^*^	.39^*^	.30^*^	.58^*^
USER	.35^*^	.38^*^	.63^*^	.41^*^	.39^*^	.53^*^
FS	.07	.06	.11	−.03	.01	.06

*Note. DRS* Disability-Related Support, *EWB* Emotional Well-Being, *FCPS* Family Communication and Problem Solving, *FI* Family Interaction, *FQOL Ttl* Family Quality of Life Total Scale, *FRAS-ASD* Family Resilience Assessment Scale–ASD Version, *FS* Family Spirituality, *PAR* Parenting, *PMWB* Physical/Material Well-Being, *USER* Utilizing Social and Economic Resources

^*^ *p* < .001

## Discussion

The current study is the first to validate the FRAS among a sample of family caregivers of children with ASD. The CFA indicated that Sixbey’s 6-factor structure did not hold. As such, we conducted an EFA, which retained 51 items across 3 factors, all of which were internally consistent. In fact, the subscale alpha values were improved from those reported in the original study [23]. Conceptually, the three factors, Family Communication and Problem Solving (FCPS), Utilizing Social and Economic Resources (USER), and Family Spirituality (FS), broadly align with Walsh’s [20] 3-domain model of family communication processes, organizational patterns, and belief systems, respectively (refer to Table 1).

Although the sub-processes proposed by Walsh did not emerge as distinct factors within our analyses, they are reflected within the retained factors. For example, family communication processes are said to include clarity and consistency, open emotional expression, and collaborative problem solving. Indeed, the majority of items within the FCPS subscale focus on collaborative problem solving, though both clarity of communication (e.g., “We understand communication from other family members”) and open emotional expression (e.g., “We feel free to express our opinions”) are certainly represented. In the current study all items from the original scale’s Maintaining a Positive Outlook and Ability to Make Meaning Within Adversity subscales loaded on factor 1 (FCPS) (standardized factor loadings ranged from .44–.74). This finding is likely related to the fact that all Maintaining a Positive Outlook items refer to optimism with regard to the family’s ability to solve problems, and are therefore very difficult to distinguish conceptually from those included within the original FCPS subscale (e.g., “We can work through difficulties as a family” versus “We can solve major problems”). This was also the case for two of the items from the original Ability to Make Meaning Within Adversity subscale, which refer to accepting that problems happen. The other item (“The things we do for each other make us feel a part of the family”) was likely retained within factor 1, as one would expect that giving time and energy to the family, openly demonstrating love and affection, collaborating in problem solving, and being attuned to others’ emotions would make members feel closely connected.

Family organizational patterns refer to a family’s use of social and economic resources, sense of connectedness, and flexibility in response to change. The FRAS-ASD USER subscale aligns with this process, as most items refer to whether families can rely on people in their community for assistance, and at a broad level, reflects one’s sense of community connectedness. The original FRAS contained a Family Connectedness subscale; however, in the current analyses, three of the items were not retained in the final solution and three were distributed across other subscales. One such item (“We show love and affection for family members”) loaded strongly (.51) on the FCPS subscale, and the other two, which referenced involvement in the community and feeling valued by friends, loaded on factor 2 (standardized factor loadings of .62 and .46, respectively). The final organizational sub-process identified by Walsh, flexibility, is only assessed in relation to problem solving (e.g., “We try new ways of working with problems” in the FCPS subscale), and is not represented within the FRAS-ASD as a distinct construct.

Finally, Walsh described family belief systems as involving transcendence and spirituality, positivity, and finding meaning from adversity. Within the FRAS-ASD, only family spirituality is assessed as a distinct sub-process (i.e., FS emerged as its own factor), with the structure being identical to that of the original tool. A limitation of this subscale, however, is that the included items portray a fairly narrow conceptualization of spirituality, with three of the four items inquiring directly about formal religion. Walsh, in contrast, highlights that families may practice spirituality in other ways, such as through participation in cultural traditions (e.g., through nature, music, or the arts). As such, it may be fruitful for future research to evaluate whether the inclusion of items broadening this conceptualization (e.g., We can express ourselves through music; Our family enjoys being outside together; We feel connected to the natural elements around us) produce a tool that better captures Walsh’s original intent. As described, the other two sub-processes (positivity and making meaning of adversity) are reflected within the FCPS subscale.

Overall, the content of the FRAS-ASD subscales align closely with research examining protective factors for families of children with ASD. Indeed, within qualitative research, caregivers highlight the importance of processes included within the FCPS subscale, specifically describing how flexibility, positivity, open communication and working together has helped them to adapt successfully to raising a child with ASD [15, 17]. Similarly, quantitative research identifies that positivity, optimism, acceptance, and confidence with regard to confronting future adversities act as buffers for maladaptive outcomes, such as parental stress, depression, and poor life satisfaction (as reviewed by Bekhet and colleagues [16]). The USER subscale content is also consistent, as families identify social support from friends, family, and the community as valued [16, 17, 23]. Although spirituality has received less attention in the literature, themes around gaining strength through faith, and from spiritual and religious practices do emerge [15–17]. Although this suggests that the FRAS-ASD is well-suited to evaluating resilience within families of children with ASD, the tool does not include items specific to this circumstance. Research identifies that families place great value on access to professionals as well as to other families of children with ASD [17], from whom they can gain knowledge and seek advice, and items related to the availability of these kinds of supports may improve the tool for this context.

The fact that the 9 sub-processes described by Walsh [20, 21] did not emerge as distinct factors is not surprising, given that one would expect at least some degree of overlap across these components. In reality, family resilience processes are not mutually exclusive, but are instead mutually influencing and reinforcing. For example, successful family problem solving involves sub-processes that cross domains, such as *flexible organizational patterns* in trying new approaches when existing ones are unsuccessful and *open communication* in order to reach a solution that is acceptable to all family members. The success of utilizing these approaches, in turn, contribute to a family’s *optimistic beliefs* about how well prepared they are to face both ongoing and future adversities. In fact, Walsh’s descriptions reflect this overlap, as making meaning from adversity, a belief system sub-process, is described in relation to the framing of family crises. This points to the inherent challenges associated with operationalizing conceptual models, as well as with measuring complex and dynamic inter-family processes. We suggest that the FRAS-ASD provides a psychometrically-sound assessment of resilience amongst families of children with ASD that is guided by Walsh’s 3-domain model.

Although this is the first study to validate this tool with a sample of caregivers of children with ASD, other validation studies have been conducted. The structure of the FRAS-ASD differs somewhat from those identified using CFA with Turkish [32] and Chinese [33] undergraduate students. Kaya and Arici [32] reported a 4-factor structure, consisting of FCPS, USER, Maintaining a Positive Outlook, and Ability to Make Meaning from Adversity. Li et al.’s [33] FRAS-C, in contrast, retained 32 items across 3 factors: FCPS, Utilizing Social Resources, and Maintaining a Positive Outlook. The observed differences are not surprising when taking into account the significant ‘family life’ differences one would expect between young adults attending university and family caregivers of children with ASD, as well as the inextricable influence of culture on resilience [53, 54]. Chew and Haase [27] conducted an EFA with adolescents (mean age = 15 years) with epilepsy in Singapore, and identified 7 factors (Meaning-Making and Positive Outlook, Transcendence and Spirituality, Flexibility and Connectedness, Resources-Community, Resources-Neighbours, Clarity and Open Emotional Expression, Collaborative Problem-Solving). We suspect that the observed structural differences also relate to cultural contexts, particularly around spirituality, connectedness, open communication, and reliance on social resources. However, their findings highlight the possibility that this tool may be utilized to attain multiple family members’ perspectives, representing an exciting extension for a field that has been dominated by maternal report.

The correlations across the FRAS-ASD and Beach Center FQOL Scale subscales provide support for convergent validity. Although FQOL and ‘family resilience’ represent distinct constructs, the Beach Center FQOL Scale was chosen given both the lack of research with this population and of established measures. It is not surprising that the strongest association was between the FCPS and FQOL Family Interaction subscale, as both refer to a family environment that supports open communication, collaborative problem solving, and demonstrations of affection. The other subscales, though related, are more conceptually distinct, and this is reflected in the observed associations. Future research may seek to examine how these two FRAS-ASD subscales converge with specific measures that purport to assess analogous constructs. Though family resilience can certainly be construed as an indicator of overall family well-being, the FRAS-ASD may best provide a measure of how well a family is utilizing various processes (i.e., positive communication patterns, reliance on social support, and religious practices) that can act as protective factors, with the potential to moderate relationships between established risk factors, such as high child behavior problems, and FQOL.

### Limitations, strengths, and future directions

The current findings should be considered within the context of the study limitations. First, the conclusions may be limited by our relatively small sample size, and may not generalize. Though the Kaiser-Meyer-Olkin measure verified the sampling adequacy for the analysis (KMO = .90) and Bartlett’s test of sphericity, χ^2^ (1431) = 6069.71, *p* < .001, indicated that correlations between items was sufficiently large for EFA, others have reported that samples of at least 300 participants are necessary [55]. Second, our sample represented a fairly ‘resource-rich’ group, as most respondents reported that they were married, well educated, and had high family incomes. The structure of the FRAS may look somewhat different for families who face more socioeconomic challenges, and future research should make efforts to include a more heterogeneous sample. The generalizability of our findings, however, are strengthened by our sample diversity with respect to reported family ethnicities, as well as the inclusion of families with children spanning a wide age range (2–35 years). Studies of family adaptation when there is a child with a disability have typically focused on the early and middle childhood years, thus neglecting the distinct challenges facing families of young adults. This developmental period can represent a significant transition for family life [56], as families are adapting to decreases in service availability, and may be negotiating changes around the structure of caregiving (i.e., responsibility may shift from parents to siblings) [57]. The fact that the FRAS-ASD has been validated with families of children with ASD ranging from early childhood to adulthood suggests that this tool may facilitate our understanding of how resilience develops and evolves across the family life cycle. Of course, however, there are myriad within-family individual factors that will influence how families experience and perceive their resilience, including those related to the vast diversity in presentation amongst those with ASD. Presence of child internalizing and externalizing difficulties, functional status, and age of diagnosis are but a few examples. Future longitudinal research would help to disentangle how resilience processes unfold in response to adversities across the life cycle of the family.

Finally, although the FRAS purports to measure ‘family’ resilience, it is important to recognize that any insights gained are representative of only one member’s perspective. This issue plagues the family adaptation literature, which is dominated by maternal report (see DeHaan et al. [11] for a discussion of methodological strategies to ameliorate this challenge). Chew and Haase’s [27] findings, however, suggest that the FRAS may be appropriate for use with multiple members, including children, which at the very least provides a more holistic picture as to how different individuals perceive inter-family dynamics. Research designs that incorporate multiple family members will best capture the “moving pictures” of family life ([58], p. 7).

## Conclusions

We have demonstrated that the FRAS-ASD provides a valid assessment of resilience amongst families of individuals with ASD, and maps to Walsh’s 3-domain theoretical model. We suggest that this tool facilitates our understanding of within-family processes that help to protect against the impact of adversity. From a clinical perspective, there is much to be learned from families who thrive despite facing considerable challenge. By encouraging family members to listen to one another, freely express their views and needs, seek out community support, and utilize constructive problem solving strategies, family resilience can be strengthened, and we can continue to move the family adaptation field away from a focus on dysfunction.

## Abbreviations

ASD: Autism Spectrum Disorder
CFA: Confirmatory Factor Analysis
CFI: Comparative Fit Index
CI: Confidence Interval
DRS: Disability-Related Support
EFA: Exploratory Factor Analysis
EWB: Emotional Well-Being
FAD: Family Assessment Device
FCPS: Family Communication and Problem Solving
FI: Family Interaction
FQOL Ttl: Family Quality of Life Total Scale
FQOL: Family Quality of Life
FRAS: Family Resilience Assessment Scale (Sixbey, 2005 version)
FRAS-ASD: Family Resilience Assessment Scale–ASD Version
FS: Family Spirituality
PAR: Parenting
PMWB: Physical/Material Well-Being
RMSEA: Root Mean Squared Error of Approximation
SRMR: Standardized Root Square Mean Residual
TLI: Tucker-Lewis Index
USER: Utilizing Social and Economic Resources.
